# Association between cortical thickness and cognitive ability in very preterm school-age children

**DOI:** 10.1038/s41598-024-52576-5

**Published:** 2024-01-29

**Authors:** Uk-Su Choi, So-Yeon Shim, Hye Jung Cho, Hyejin Jeong

**Affiliations:** 1 Medical Device Development Center, Daegu-Gyeongbuk Medical Innovation Foundation, Daegu, South Korea; 2 Division of Neonatology, Department of Pediatrics, School of Medicine, Ewha Womans University, Seoul, South Korea; 3 Division of Neonatology, Department of Pediatrics, Gil Medical Center, Gachon University College of Medicine, Incheon, South Korea; 4 Neuroscience Convergence Center, Institute of Green Manufacturing Technology, Korea University, Seoul, South Korea

**Keywords:** Paediatric research, Developmental biology, Neuroscience, Medical research

## Abstract

Very preterm children, born before 32 weeks of gestation, are at risk for impaired cognitive function, mediated by several risk factors. Cognitive impairment can be measured by various neurodevelopmental assessments and is closely associated with structural alterations of brain morphometry, such as cortical thickness. However, the association between structural alterations and high-order cognitive function remains unclear. This study aimed to investigate the neurodevelopmental associations between brain structural changes and cognitive abilities in very preterm and full-term children. Cortical thickness was assessed in 37 very preterm and 24 full-term children aged 6 years. Cortical thickness analysis of structural T1-weighted images was performed using Advanced Normalization Tools. Associations between cortical thickness and the Wechsler Intelligence Scale for Children were evaluated by regression analysis based on ordinary least square estimation. Compared with full-term children, very preterm children showed significant differences in cortical thickness, variously associated with cognitive abilities in several brain regions. Perceptual reasoning indices were broadly correlated with cortical thickness in very preterm and full-term children. These findings provide important insights into neurodevelopment and its association with cortical thickness, which may serve as a biomarker in predictive models for neurodevelopmental diagnosis of high-order cognitive function.

## Introduction

Very preterm children, born before 32 weeks of gestation^1^, commonly show impaired cognitive function, including learning and memory, lower academic performance, behavioral problems, and lower intelligence, which persist throughout childhood and young adulthood^2–8^. Early school age, starting from 6 years, is a critical period during which children start robust learning and formal schooling commences^9^.

Cortical thickness studies using magnetic resonance imaging (MRI) have shown structural alterations in very preterm children compared with age-matched full-term controls^10,11^. These include alterations in cortical thickness in several brain areas, including the parietal and temporal cortices^7,12–15^. A decline in cortical thickness has been shown from the early stages of childhood through to adolescence, accompanied by the elimination of unnecessary neurons and synapses^14^, creating efficient synaptic connections. These structural changes have been associated with cognitive and behavioral impairment during childhood and adolescence^16–18^.

Most studies have focused on differences in brain morphometry, such as volume and cortical thickness, between preterm and full-term children^19–21^. Many studies have evaluated the associations between morphometry and developmental assessments, such as language skills and motor function^16–18^. Other studies have described higher cognitive function in intelligence or working memory domains in full-term children; however, most studied participants older than 6 years^10,11,22–24^. Although the age around 6 years has been demonstrated as a critical period in cortex development and cognitive developmental aspects^25–28^, there have been few studies on children of this age.

In this study, we aimed to estimate the associations between structural alterations and cognitive abilities based on neurodevelopmental assessments in very preterm and full-term children at 6 years of age.

## Results

### Clinical characteristics and neurodevelopmental outcomes

The demographic and clinical characteristics of the participants are presented in Table 1. The average gestational age (GA) of the very preterm children was 27.5 ± 2.4 weeks, and the average birth weight was 1082.16 ± 346.84 g. All the full-term children were born after 37 weeks of gestation and weighed > 2500 g at birth. There were no significant differences in the age of the participants at the time of the MRI scan, or in the sex distribution between the very preterm group (mean age: 76.2 ± 3.7 months, 23 males) and the full-term group (mean age: 80.0 ± 3.2 months, 14 males). Both groups performed at or above normal limits on all assessments (mean standardized score = 100 with a standard deviation of 15). The full-term group scored higher than the very preterm children on all included subscales of the Wechsler Intelligence Scale for Children, fourth edition (WISC-IV). The very preterm group scored higher than the full-term group in Child Behavior Checklist (CBCL) scores for social problems, attention problems, rule-breaking, aggressive behavior, externalizing problems, and total problems. The results of the neurodevelopmental assessments are described in Table 2.

**Table 1:** Demographics and clinical characteristics.

Characteristic	Preterm (n = 37)	Full-term (n = 24)	*P* value
Gestational age (weeks)	27.5 ± 2.38	39.5 ± 1.40	< 0.001
Birth weight (g)	1082.16 ± 346.84	3346.67 ± 384.35	< 0.001
Male sex	23 (62.2)	14 (58.3)	0.570
Age at study (months)	76.20 ± 3.74	80.04 ± 3.20	0.180
Intraventricular hemorrhage (grade 1)	18 (48.6)		
Respiratory distress syndrome	20 (54.1)		
Patent ductus arteriosus requiring medical or surgical management	22 (59.5)		
Bronchopulmonary dysplasia (≥ moderate)	12 (32.4)		
Necrotizing enterocolitis (≥ stage 2)	7 (18.9)		
Proven sepsis	3 (8.1)		

Data are presented as the mean ± SD values for continuous variables and *n* (%) values for categorical variables.

*SD* standard deviation.

**Table 2:** Comparison of neurobehavioral outcomes.

Test	Preterm (n = 37)	Full-term (n = 24)	*P* value
WISC-IV			
Verbal comprehension index	89.76 ± 15.74	99.48 ± 12.69	0.029
Perceptual reasoning index	87.46 ± 16.13	101.52 ± 13.10	0.001
Working memory index	86.89 ± 17.10	96.05 ± 10.77	0.019
Processing speed index	86.59 ± 18.11	98.86 ± 15.74	0.027
FSIQ	84.08 ± 15.69	97.81 ± 10.97	0.001
CBCL			
Anxious/depressed	56.81 ± 5.31	55.25 ± 7.64	0.331
Withdrawn/depressed	56.41 ± 6.28	53.92 ± 4.38	0.122
Somatic complaints	55.95 ± 6.51	55.08 ± 6.23	0.591
Social problems	61.30 ± 9.74	54.67 ± 6.81	0.028
Thought problems	58.59 ± 6.83	55.58 ± 4.76	0.132
Attention problems	57.84 ± 8.35	52.58 ± 4.54	0.008
Rule-breaking	57.78 ± 6.45	54.42 ± 3.92	0.021
Aggressive behavior	58.43 ± 8.88	52.33 ± 3.75	0.002
Internalizing problems	56.57 ± 6.61	52.17 ± 9.46	0.058
Externalizing problems	58.11 ± 11.84	49.75 ± 6.37	0.015
Total problems	58.51 ± 8.78	50.50 ± 8.61	0.008

Data are presented as the mean ± SD values for continuous variables and *n* (%) values for categorical variables.

*SD* standard deviation, *WISC-IV* Wechsler intelligence scale for children, fourth edition, *FSIQ* full-scale intelligence quotient, *CBCL* child behavior checklist.

### Results of cortical thickness analysis

**Figure 1:**
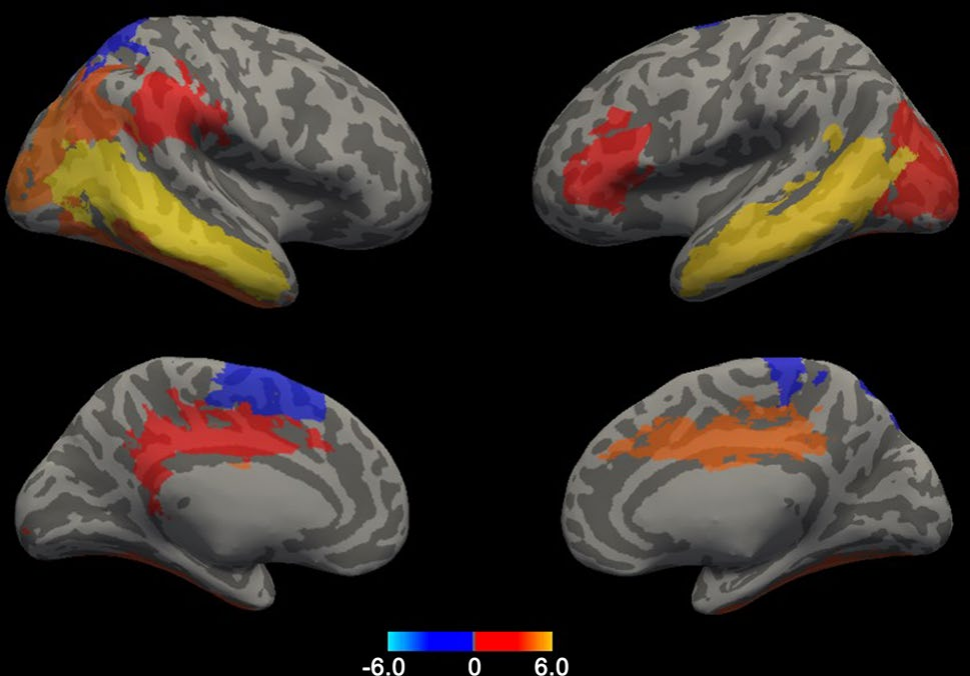
Differences in cortical thickness between very preterm and full-term children. Areas with significant differences are shown in color, with red to yellow representing a thicker cortex and dark to light blue representing a thinner cortex in the very preterm children compared with the full-term children. Threshold false discovery rate (FDR), *P* < 0.05.

In the cortical thickness analysis, the cortex of very preterm children were found to be significantly thinner than that of full-term children in the left supplementary motor area, right superior parietal gyrus, and right paracentral lobule. Conversely, the cortex of very preterm children was significantly thicker than that of full-term children in the following 13 regions of interest: the triangular part of the inferior frontal gyrus and posterior cingulate gyrus in the left hemisphere; the median cingulate gyrus, middle occipital gyrus, fusiform gyrus, and middle temporal gyrus bilaterally; and the supramarginal gyrus, angular gyrus, and inferior temporal gyrus in the right hemisphere (Fig. 1, Table 3).

**Table 3:** Anatomical areas with significant differences (*P* < 0.05) in mean cortical thickness between preterm and full-term children.

Anatomical regions	Hemisphere	Preterm (n = 37)	Full-term (n = 24)	*P *value
Preterm > full-term				
Inferior frontal gyrus (triangularis)	L	1.26 ± 0.09	1.19 ± 0.12	0.007
Median cingulate gyrus	L	2.16 ± 0.15	2.04 ± 0.14	0.004
Median cingulate gyrus	R	2.26 ± 0.15	2.11 ± 0.12	< 0.001
Posterior cingulate gyrus	L	1.66 ± 0.23	1.51 ± 0.12	0.004
Middle occipital gyrus	L	1.54 ± 0.17	1.40 ± 0.15	0.002
Middle occipital gyrus	R	1.78 ± 0.20	1.57 ± 0.14	< 0.001
Fusiform gyrus	L	2.26 ± 0.15	2.11 ± 0.17	0.001
Fusiform gyrus	R	2.33 ± 0.14	2.17 ± 0.20	< 0.001
Supramarginal gyrus	R	1.34 ± 0.17	1.23 ± 0.11	0.008
Angular gyrus	R	1.65 ± 0.16	1.50 ± 0.13	< 0.001
Middle temporal gyrus	L	1.58 ± 0.17	1.37 ± 0.11	< 0.001
Middle temporal gyrus	R	1.65 ± 0.19	1.39 ± 0.15	< 0.001
Inferior temporal gyrus	R	1.62 ± 0.13	1.49 ± 0.15	< 0.001
Preterm < full-term				
Supplementary motor area	L	1.44 ± 0.15	1.57 ± 0.16	0.002
Superior parietal gyrus	R	1.20 ± 0.14	1.32 ± 0.21	0.005
Paracentral lobule	R	0.89 ± 0.15	1.01 ± 0.19	0.009

Data are presented as the mean ± SD values for continuous variables.

*SD* standard deviation, *L* left, *R* right.

### Association between cortical thickness and neurodevelopmental assessment results

**Figure 2:**
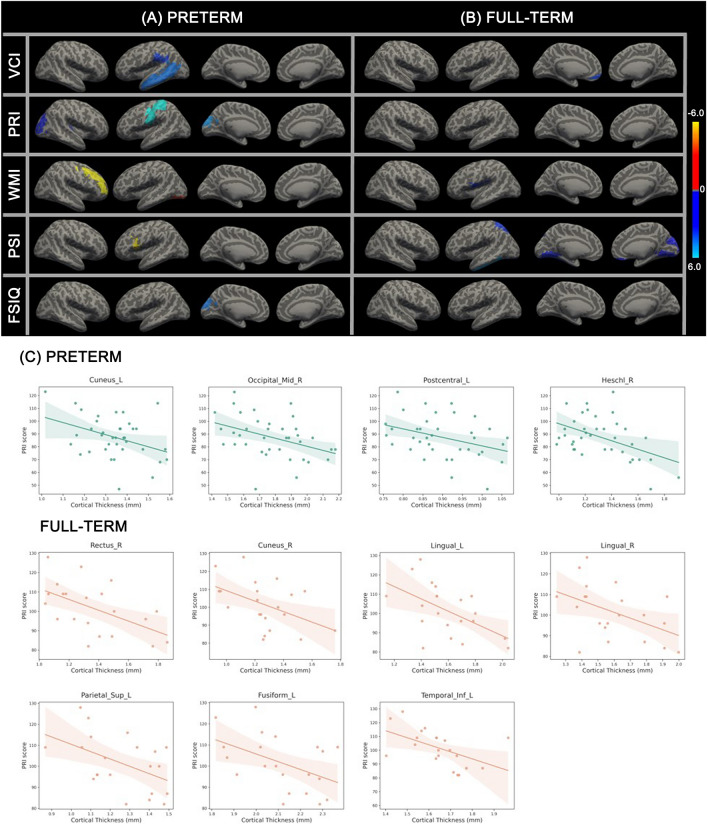
Regression analysis between cortical thickness and the Wechsler Intelligence Scale for Children, fourth edition (WISC-IV) indices in (**A**) very preterm and (**B**) full-term children. Red areas show positive coefficients, and blue areas show negative coefficients. (**C**) Association between the perceptual reasoning index (PRI) scores and cortical thickness in very preterm and full-term children. Threshold false discovery rate (FDR), *P* < 0.05.

Figure 2 and Table 4 describe the five WISC-IV scores and their respective correlations with regional cortical thickness. In very preterm children, the Verbal Comprehension Index (VCI) scores showed a negative correlation with cortical thickness in the left supramarginal gyrus (coefficient = − 35.80, *P* = 0.032) and left middle temporal cortex (coefficient = − 43.15, *P* = 0.004). The Perceptual Reasoning Index (PRI) scores showed a negative correlation with cortical thickness in the left cuneus (coefficient = − 47.32, *P* = 0.021), postcentral gyrus (coefficient = − 66.49, *P* = 0.028), right middle occipital cortex (coefficient = − 31.73, *P* = 0.014), and Heschl’s gyrus (coefficient = − 33.38, *P* = 0.003). The Working Memory Index (WMI) scores showed a positive correlation with cortical thickness in the right middle frontal cortex (coefficient = 70.32, *P* = 0.021) and the left inferior occipital cortex (coefficient = 34.10, *P* = 0.005). The Processing Speed Index (PSI) scores showed a positive correlation with cortical thickness in the left opercular part of the inferior frontal gyrus (coefficient = 58.02, *P* = 0.014). The Full-Scale Intelligence Quotient (FSIQ) scores showed a negative correlation with cortical thickness in the left cuneus gyrus (coefficient = − 43.64, *P* = 0.029) (Fig. 2A, C). In full-term children, significant negative correlations with cortical thickness were observed only for the VCI, PRI, and WMI scores. The VCI scores showed negative correlations with cortical thickness in the left medial orbitofrontal cortex (coefficient = − 36.47, *P* = 0.029). The PRI scores showed negative correlations with cortical thickness in the left lingual gyrus (coefficient = − 32.04, *P* = 0.011), superior parietal cortex (coefficient = − 34.98, *P* = 0.027), fusiform gyrus (coefficient = − 36.73, *P* = 0.033), inferior temporal cortex (coefficient = − 51.00, *P* = 0.015), right rectus gyrus (coefficient = − 27.93, *P* = 0.012), cuneus gyrus (coefficient = − 29.27, *P* = 0.033), and lingual gyrus (coefficient = − 28.15, *P* = 0.034). The WMI scores showed a negative correlation with cortical thickness in the left rolandic operculum (coefficient = − 33.33, *P* = 0.025) (Fig. 2B, C).

**Table 4:** Significant correlations between cortical thickness and neurodevelopmental assessment index in the very preterm group and full-term group.

Region	Brain lobe	VCI	PRI	WMI	PSI	FSIQ
Preterm						
Right middle frontal	Frontal	ns	ns	70.32	ns	ns
Left frontal inferior operculum	Frontal	ns	ns	ns	58.02	ns
Left cuneus gyrus	Occipital	ns	− 47.32	ns	ns	− 43.64
Right middle occipital	Occipital	ns	− 31.73	ns	ns	ns
Left inferior occipital	Occipital	ns	ns	34.10	ns	ns
Left postcentral gyrus	Parietal	ns	− 66.49	ns	ns	ns
Left supramarginal gyrus	Parietal	− 35.80	ns	ns	ns	ns
Right Heschl’s gyrus	Temporal	ns	− 33.38	ns	ns	ns
Left middle temporal	Temporal	− 43.15	ns	ns	ns	ns
Full-term						
Left Rolandic operculum	Frontal	ns	ns	− 33.33	ns	ns
Left medial orbitofrontal cortex	Frontal	− 36.47	ns	ns	ns	ns
Right rectus gyrus	Frontal	ns	− 27.93	ns	ns	ns
Right cuneus gyrus	Occipital	ns	− 29.27	ns	ns	ns
Left lingual gyrus	Occipital	ns	− 32.04	ns	ns	ns
Right lingual gyrus	Occipital	ns	− 28.15	ns	ns	ns
Left superior parietal	Parietal	ns	− 34.98	ns	ns	ns
Left fusiform gyrus	Temporal	ns	− 36.73	ns	ns	ns
Left inferior temporal	Temporal	ns	− 51.00	ns	ns	ns

Data are presented as coefficients of the very preterm and full-term group among brain regions. A *P* value of < 0.05 was considered significant.

*VCI* verbal comprehension, *PRI* perceptual reasoning, *WMI* working memory, *PSI* processing speed, *FSIQ* full-scale intelligence quotients, *ns* not significant.

## Discussion

This study investigated the brain morphometry-cognitive function relationship in a sample of 37 very preterm and 24 full-term children by calculating cortical thickness and assessing cognitive abilities. We found significant differences in cortical thickness in several brain regions between the very preterm and full-term groups at 6 years of age. Compared with that in full-term children, the very preterm children showed a significantly thinner cortex in the supplementary motor area of the left hemisphere and the superior parietal gyrus and paracentral lobule of the right hemisphere. The very preterm children also had a significantly thicker cortex globally; bilaterally in the frontal, middle occipital cortex, fusiform gyrus, and middle temporal cortex; and in the right parietal cortex, consistent with previous reports^10,11^. Two different intraventricular hemorrhage (IVH) groups (no IVH and grade 1 IVH) included in the very preterm children showed no significant differences in structural morphometry in those brain regions. This aligns with previous research which shows lower grade IVH groups have neurodevelopmental profiles similar to healthy participants^29,30.^

The development of cortical thickness varies across brain regions due to differential synaptic development and the complexity of laminar architecture^8,31^. Some brain regions have shown an inverted U-shaped progression in cortical thickness, with an increase in thickness up to 10 years of age, followed by a decline^32^. Other studies have reported a linear decline or quadratic trajectories of cortical thickness after 8 years of age^33^. The increase in cortical thickness during puberty and adolescence can be attributed to synaptic reorganization^34^. The subsequent decrease in cortical thickness is predominantly due to intracortical myelination and increased axonal caliber, i.e., white matter (WM) development^35–37^. We previously demonstrated delayed development of the entire WM in preterm neonates compared to full-term controls; however, at 1 year of age, WM development, other than that of the corpus callosum, had reached the development level of the full-term controls^38,39^. At 6 years of age, there was no significant difference between the groups^40^. Our results are consistent with previous reports showing that primary sensory-motor areas reach peak cortical thickness earlier than other brain regions^41^. In our very preterm children, decreased cortical thickness was found in the motor areas and adjacent parietal regions^42^, whereas increased cortical thickness was observed in the occipital, temporal, and frontal regions. Due to the variability in the timing of peak cortical thickness across the brain regions and the delayed cortical changes found in very preterm children compared with full-term children, significant differences in cortical thickness were observed at 6 years of age.

We also found significant relationships between structural alterations and neurodevelopmental measurements. In our study, both very preterm and full-term children showed negative associations in most brain regions, indicating that most cortices had passed the peak point at the age of 6 years. However, unlike full-term children, the very preterm children showed positive associations with the WMI scores in the frontal region (the right middle frontal and the left frontal inferior operculum) and the occipital region (the left inferior occipital region). The occipital region in the very preterm children has been reported to show a significant relationship with working memory^43^. This positive association could be explained by relatively late cortical development in the frontal and some occipital regions, compared with other brain regions^9^. Full-term children have faster cortical development than the very preterm children, and decreased cortical thickness after the peak reflects grey matter (GM) maturation induced by the reduction of neuronal cells or synoptic processes as a normal part of aging or WM maturation due to the myelin coating of fibers^44,45^. Assuming that structural and functional (cognitive) development is linear, it is natural for functional measurements to increase as cortical thickness decreases after passing the peak, showing cortical development and negative regression with functional measurements. Among the five neurodevelopmental assessments, the PRI scores commonly showed significant associations in the occipital, parietal, and temporal regions, and in the case of the full-term children, in the frontal region (the right rectus). The PRI is a widely used measure for higher-order cognitive functions, such as interpreting and organizing visual information to solve problems^46^. The index could also be an important biomarker reflecting developmental ability in neurodevelopmental disorders, such as autism spectrum disorder^47^. In both very preterm and full-term children, a higher PRI score correlated with decreased cortical thickness (Fig. 2C). The PRI could be a significant indicator for understanding neurodevelopment, and may serve as a biomarker in predictive models for both very preterm and full-term children. These findings suggest a discrete association of structural aspects with brain function in very preterm and full-term children.

However, some limitations of this study should be acknowledged. First, owing to the absence of longitudinal datasets, there is no clear support for our explanation of the differences in cortical thickness. The detailed trajectory of brain morphometry across childhood can provide evidence for the guidance of neurodevelopmental status^20,48^. Second, the sample size was relatively small compared to previous studies, despite the significant results obtained. Therefore, future studies with larger sample sizes are needed to confirm the generalizability of our findings. Finally, further investigation of the association between neurobehavioral outcomes and structural morphometry across different neurodevelopmental groups should be conducted. Although we found some significant differences in behavioral problems between preterm and full-term groups^49^, multiple neurobehavioral measurements, including WISC-IV, can play a crucial role in enhancing our understanding of neurodevelopment.

We identified differences in cortical thickness in very preterm children aged 6 years of age compared with full-term children of the same age. In both groups, cortical thickness was significantly correlated with higher-order cognitive functions. These findings clarify the pathophysiology of cortical thickness and its association with neurodevelopment in very preterm children, demonstrating the long-term impact of very preterm birth on structural and cognitive function.

## Methods

### Participants

Between December 2016 and April 2019, a total of 65 children (41 very preterm children and 24 full-term children) were recruited and underwent MRI brain scans at the term-equivalent age. The very preterm group was recruited at 6 years of age, comprising children born preterm ≤ 32 weeks GA and admitted to the level IV neonatal intensive care unit at Gachon University Hospital between 2010 and 2013. The very preterm group included 18 children with grade I IVH and 19 children with no IVH. The data of four children in the very preterm group was discarded due to excessive motion artifacts. The control group included full-term children (≥ 37 weeks GA at birth). MRI scans were performed either on the same day or within 1 week of the neurodevelopmental assessment. The parents of all the children submitted written informed consent. Ethical approval was obtained from the Institutional Review Board of Gachon University Gil Medical Center (GBIRB2016-239), and all experiments were performed in accordance with the tenets of the Declaration of Helsinki.

### Neurodevelopmental assessment

Neurodevelopmental assessments were conducted using the WISC-IV, and the CBCL. The WISC-IV provides full-scale intelligence quotients, which represent overall cognitive abilities, and four indices based on specific cognitive domains: verbal comprehension, perceptual reasoning, working memory, and processing speed. The CBCL assesses children’s behavioral problems.

### MRI acquisition

A 3.0-T Siemens scanner (Verio, Siemens, Germany) and a Siemens matrix coil were used. T1-magnetization-prepared rapid gradient-echo (MPRAGE) images of all participants were obtained under the supervision of an attending pediatrician. The T1-MPRAGE imaging parameters used were as follows: repetition time = 1900 ms; echo time = 2.93 ms; flip angle = 8°; pixel bandwidth = 170 Hz/pixel; matrix size = 256 × 208; field-of-view = 256 mm; number of excitations = 1; slice thickness = 1 mm; total acquisition time = 4 min 9 s. The MRI scans were scheduled around the child’s natural nap time to encourage successful scanning without using sedatives. In case of inability to fall asleep naturally, a low dose of chloral hydrate (30 mg/kg) was administered orally.

### Cortical thickness analysis

A cortical thickness analysis of the structural T1-weighted (T1w) images was performed using a customized shell script, the antsCorticalThickness pipeline, based on Advanced Normalization Tools^50^ which are widely adopted for the preprocessing of structural MRI. The script consisted of several preprocessing steps, segmentation of brain tissue, and a volume-based cortical thickness strategy called diffeomorphic registration-based cortical thickness (DiReCT)^51,52^ that has shown sufficient performances in reproducibility or reliability with large-scale data^53,54^. The preprocessing steps included inhomogeneity correction of the T1w images and brain extraction for skull stripping. After the preprocessing steps, the corrected T1w images were divided into three brain tissues: cerebrospinal fluid (CSF), GM, and WM, using the Atropos algorithm^52^. As a final step, a cortical thickness map was constructed from the segmented brain tissue images for each participant using the DiReCT method, which is a widely used method based on volumetric information of diffeomorphic mapping from segmented tissues. Our code for running the cortical thickness analysis was as follows: the files ct1w.nii.gz, t1w_templated.nii.gz, and prob_template.nii.gz represent the corrected T1-weighted images, the template of the T1-weighted image, and the probability maps of the GM, WM, and CSF, respectively. The syntax in the code has the following meanings: -d represents a data type (either two- or three-dimensional), -a is used for an input image, -e is used for a template image, -m stands for a probability map, -p stands for tissue probability priors, and -o is the output prefix.

- antsCorticalThickness.sh \
-d 3 \
-a ct1w.nii.gz \
-e t1w_template.nii.gz \
-m prob_template.nii.gz \
-p prob%02d.nii.gz \
-o ct_.

### Statistical analyses

Demographic and neurodevelopmental assessment scores were compared between very preterm and full-term children via two-sample *t* tests using the Statistical Package for the Social Sciences, version 22.0, software (IBM Corporation, Armonk, NY, USA). The values were represented as counts (%) or means ± standard deviations. Categorical variables were compared using the chi-square test, and continuous variables were compared using the independent Student’s *t* test for univariate analyses. The differences in regional cortical thickness between the two groups were evaluated using two-sample t-tests. Statistical measures with *P* values < 0.05 were considered statistically significant. To correct for multiple comparisons of the CBCL scores, the significance level was set at *P* < 0.004, using the Bonferroni correction (11 comparisons). In addition, linear regression analysis was performed to investigate the correlations between cortical thickness and the five indices of the WISC-IV. This analysis was based on ordinary least square estimation, with a significance level set at *P* < 0.05, using the statsmodels package in Python.

## Data Availability

The data that support the findings of this study are available from the corresponding author upon reasonable request.
